# Mesothelioma: Hippo pathway as a target, lessons from COMMAND

**DOI:** 10.18632/oncotarget.27018

**Published:** 2019-06-18

**Authors:** Dean A. Fennell, Essa Y. Baitei

**Affiliations:** ^1^ University of Leicester and University Hospitals of Leicester, Leicester Mesothelioma Research Programme, Leicester Cancer Research Centre, Leicester, Leicestershire, UK

**Keywords:** hippo pathway, focal adhesion kinase, mesothelioma, defactinib, NF2

Mesothelioma is an incurable cancer caused by exposure to asbestos. Several countries have witnessed a growth in incidence of epidemic proportions over the last two decades, and approved treatment is limited to front-line chemotherapy [1]. The recently reported COMMAND trial [2] was a robustly designed, hypothesis-driven, double blind randomized phase II trial. This study evaluated the efficacy of the focal adhesion kinase inhibitor Defactinib versus placebo as a maintenance therapy in patients with Merlin negative mesothelioma [2]. COMMAND is one of the few reported randomized trials in mesothelioma that have incorporated prospective molecular stratification, another being the ADAM study [3].

COMMAND was negative, despite hopes of recapitulating preclinically observed synthetic lethality in the context of NF2 mutation [4], as well as targeting of the stem cell compartment [5]. Merlin is encoded by NF2 which undergoes positive selection in mesothelioma [6], a gene affected by somatic copy number alterations, fusions and epigenetic suppression [7]. In COMMAND, detection of aberrant Merlin, was therefore determined using immunohistochemistry to minimize false negative stratification that could occur through exclusively genetic screening.

**Figure 1:**
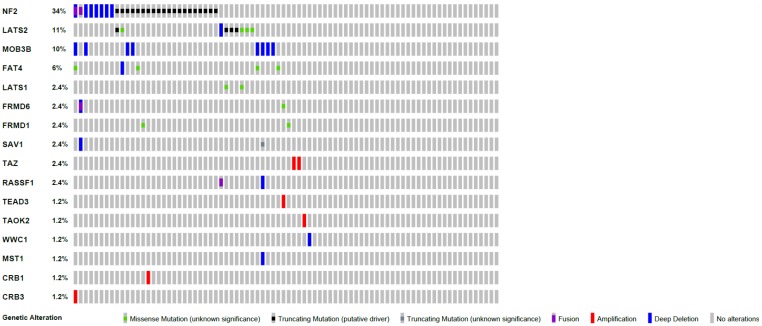
Heatmap summarizing hippo pathway mutations across 82 patients in the tumour genome atlas cohort [8, 15, 16].

Since the initiation of the COMMAND trial, comprehensive genomic studies have revealed extensive genetic disruption of hippo signaling in mesothelioma involving multiple components of this tumor suppressor pathway [7, 8]. In the Tumour Genome Atlas cohort for example, 51% of mesotheliomas harbour at least one somatic alteration in the Hippo pathway, with more than one aberration occurring in 19.5% (Figure 1). These genetic alterations involve, in addition to NF2, LATS2 (a positively selected gene), MST1, LATS1, SAV1, and RASSFA1, all capable of phenocopying loss of NF2- summarized in figure 1 [9, 10]. In hindsight, patient enrichment for just one of these components may have sampled only a proportion of “sensitive” phenotypes, limiting the ability to detect a signal.

A phase II window of opportunity trial involving defactinib monotherapy in patients prior to surgical resection demonstrated a promising 80% disease control and 13% response rate [11]. Interestingly, responses were not limited to Merlin-negative mesotheliomas. As the hippo pathway converges on YAP/TAZ-TEAD driven transcription [12], pharmacological disruption of the YAP/TEAD complex could provide a strategy for targeting hippo defective mesothelioma irrespective of the specific pathway defect [13].

After the initiation of the COMMAND trial, another important insight into FAK biology came to light. FAK plays a crucial role in regulating the tumour microenvironment via augmentation of Treg abundance [14]. Inhibiting FAK drives a CD8 host response with evidence of reduced Tregs both preclinically and clinically [11, 14]. This finding has been translated into a Cancer Research UK phase 1 clinical trial combining defactinib with pembrolizumab in patients with unselected, relapsed mesothelioma (NCT02758587).

In summary, targeting the Hippo pathway remains a potentially promising strategy for controlling mesothelioma. Widening the net to capture more hippo deficient phenotypes through the use of comprehensive *hippo defective biomarkers,* or directly antagonizing YAP/TEAD transcription may provide next-generation approaches to phenocopying this important tumor suppressor pathway in mesothelioma.
